# Investigation of the optimal timing and amount of fuel injection on the efficiency and emissions of a diesel engine through experimentation and numerical analysis

**DOI:** 10.1016/j.heliyon.2024.e38790

**Published:** 2024-10-02

**Authors:** Zuhair Aldarwish, Mohammad Hossein Aghkhani, Hassan Sadrnia, Javad Zareei

**Affiliations:** Department of Biosystems, Faculty of Agriculture, Ferdowsi University of Mashhad, Iran

**Keywords:** Experimental results, Fuel injection timing, Exhaust emissions, Engine performance, Computational fluid dynamics

## Abstract

The purpose of this study is to investigate the performance, in-cylinder combustion, and emissions of the MTE660E heavy-duty diesel engine using 3D CFD simulation. The CFD simulation model based on AVL FIRE software was developed to numerically investigate the performance, in-cylinder combustion, and emissions of the MTE660E engine. The AVL model was validated against empirical data. The 6-cylinder MTE660E engine was operated under a constant excess air ratio of 1.75 and at 1600 rpm engine speed. The geometry and lattice design of the MTE660E engine were simulated to provide a computationally efficient approach. The AVL model was validated against experimental data and the error between the measured and calculated value of combustion characteristics and emissions was acceptable (R2> 90 %), error <5.6 %). Design Expert software was used to optimize the dependent variables (peak chamber temperature, brake mean effective pressure, engine torque, engine thermal efficiency and emissions of NOx) according to the studied variables (injection time and fuel injection quantity) based on response surface methodology. The results show that the recommended injecting time of 342 °C and the specific amount of fuel lead to better combustion efficiency, resulting in high engine performance and low emissions. The specific fuel consumption was highest at 2200 rpm with a 50 % load, while the lowest SFC level was observed at 1750 rpm with a 100 % load. The maximum value of NOx emissions was observed at full load. The findings show that the optimization of the injection timing and fuel injection quantity can effectively enhance the performance of the MTE660E engine. The study provides valuable insights into improving combustion efficiency, resulting in greater performance and reduced emissions without requiring expensive overhaul. It has potential applications in automotive and commercial transportation industries, thereby reducing fuel consumption and emissions.

## Highlights

• Effect of Injection Timing and Diesel Fuel Mass on Efficiency and Emissions.
• The AVL Fire engine simulation software.
• Optimal injection timing and fuel quantity improve combustion efficiency.
• Optimal injection timing and fuel quantity reduce exhaust emissions.

**Table:** Nomenclature

*CFD*	*Computational Fluid Dynamics*	*HC*	*Hydrocarbon Concentration*
*AVL*	*An automatic Vehicle Locator*	*CI*	*Compression Ignition*
*FIT*	*Fuel Injection Timing*	*CA*	*Clank Angle*
*SFC*	*Specific Fuel Consumption*	*TDC*	*Top Dead Centre*
*SE*	*Soot Emission*	*BDC*	*Bottom Dead Centre*
*EGT*	*Exhaust Gas Temperature*	*BE*	*Braking torque*
*IVC*	*Intake Valve Closing*	*PCT*	*Peak Chamber Temperature*
*IVO*	*Intake Valve Opening*	*BMEP*	*Brake mean effective pressure*
*CO*	*Carbone Oxide*	*FTE*	*Engine thermal efficiency*
*NO*	*Nitrogen Oxide Concentration*	*HC*	*Hydrocarbon Concentration*

## 1 Introduction

As a result already shown in numerous researches, the performance and resulting emissions of diesel engines depends on the critical parameters of the fuel injection time and amount. During the past decades, with the increase in environmental concerns, the formation and release of pollutants are the main focuses of the current investigations to monitor the diesel engines performance. In this context, strict government regulations have been established to monitor emission of these pollutants from vehicles, and new technologies have been developed to implement these regulations, such as implementing nano additives to the diesel fuel [1]. In addition to pollutants from natural sources, man-made pollutants from human activities pose hazards and potential risks to the environment and human health. With the growth of societies and the increase in the volume of these pollutants in recent years, it has become a significant problem in both developed and developing countries [2]. The sources of these pollutants can be considered as combustion processes in IC (Internal Combustion) engines, which is one of the major contributors to air pollution. In the past half century, IC engines have evolved significantly in terms of geometry and design, with improved performance and reduced emissions as critical priorities in the automotive, industrial, and agricultural machinery industries. In recent years, much research work in both quantity and quality of internal combustion engines, has developed drastically, with a focus on addressing the factors that affect engine performance. Computer and theoretical simulations play an essential role in supporting this shift in approach to optimize engine performance and reduce emissions. One of the advantages of computer modeling is to reduce costs, achieve rapid results, plan production and laboratory work, etc. With the increasing focus on fuel economy, global warming and environmental issues, the use of direct injection diesel engines in agricultural and industrial applications has been widely adopted. Because of the need to optimize engine designs to reduce emissions and improve performance, a better understanding of the combustion process of these engines is essential. The amount and timing of fuel injection are important parameters for improving engine performance and combustion behavior. There is an increasing amount of research on these parameters to understand their impact on engine behavior and performance, which can inform the design of future engine models [3,4]. The state and condition of fuel injection in a diesel engine are critical factors that have received considerable attention from designers, manufacturers and researchers in the industry. It is well known that important fuel injection factors that are considered to affect engine performance include spray tip design, number, diameter, and geometry of injection holes, fuel injection pressure, injection start, injection rate shape, and injection duration [[5], [6], [7], [8]]. Most engine manufacturers and researchers currently focus on optimizing the combustion chamber shape and modifying the in-cylinder air flow and pressure to improve engine performance. An optimal number of injection holes at the higher injection pressure mainly resulted in the required energy for improved fuel-air mixture, combustion efficiency and emissions [9]. With an improved fuel injection system, it's possible to optimize both the amount of fuel and its injection timing according to the changes of engine speed and load [10,11].The diesel combustion process is highly dependent on fuel injection parameters such as rapid and homogeneous mixture between the injected fuel and air, and proper control of the fuel spray process is essential for higher performance and lower emissions of diesel engines [12,13]. Studies show that optimized injection timing plays an important role in compression ignition engines fueled with different fuels and blends [14]. Investigation on the effect of fuel injection strategies in a modern diesel engine using oxygenated fuel blending indicates that post-injection strategy reduces number of primary particles and radius of soot agglomerates [15]. On the other hand, dual fuel engines which use diesel and other fuels such as gasoline have been studied by various researcher, investigation on a PPCI Engine (partially premixed combustion ignition) with different blends of gasoline–diesel shows a significant reduction of NOx formation and PM emissions [16]. Another study on diesel engines fueled with diesel and E85 shows that cooled EGR can improve engine torque while used with PPCI combustion strategy [17].

One of the key issues in the future diesel engine development are to achieve the engine pollutant reduction such as; particulate pollutants, sulfur oxides (SOx) and nitrogen oxides (NOx) while working in opposition to each other [18]. Despite various research activities for investigating the reduction indices of each of these pollutants, many of these interventions are only able to reduce one of them, resulting in an increase in the other [19,20]. In heavy duty diesel engines, it can be challenging to optimize the combustion chamber geometry for homogeneous air-fuel mixture. The design and modification of the combustion chamber leads to reduce the mixture fraction gradient and maintain the atomization and combustion efficiency, resulting in higher engine performance, especially under different operating conditions [21]. Many methods have been developed by researchers, discussed in this paper, focusing on the possible strategies for improving fuel-air mixture homogeneity with early fuel injection inside the diesel engine combustion chamber [22]. However, the potential drawbacks associated with this approach include the lack of precise ignition timing control, which ultimately affects engine performance at different speeds and loads [23]. The changes in injection strategy and timing result in a change in flow pattern, which affects in-cylinder conditions [24]. The application of a combination of these scientific approaches to internal combustion engines has been limited to light applications and laboratory conditions. However, focusing on fuel injection characteristics and optimizing these parameters can play a significant role in improving industrial engine performance. The combustion and emission characteristics of diesel engines are modified by changing the injection strategies, which affects the engine performance and power [[25], [26], [27]]. Study on a compression ignition engine fueled with rapeseed methyl ester shows that fuel injection pressure significantly impacts soot nanoparticles size distribution [28]. Therefore, conducting further researches are essential to offer useful insights and comments on effect of injection timing and variable amount of fuel mass, as well as ignition delay on both engine performance and emissions [[29], [30], [31], [32], [33], [34]].

The primary objective of this study is to explore the impact of varying fuel injection quantities and timings on the performance and emissions of the MTE660E test engine. This investigation utilizes a cutting-edge approach, employing a CFD 3D model developed using AVL FIRE software, alongside analytical computer simulations. The intricate geometry and lattice of the MTE660E engine have been meticulously crafted for this purpose. Through numerical simulations conducted at 1600 rpm and an excess air ratio of 1.75, the flow dynamics within the cylinder have been comprehensively analyzed and cross-validated with empirical data. Leveraging an optimization tool and regression model derived from simulation outcomes, the study delves into optimizing injected fuel mass and timing. Renowned for their robustness and efficiency, MTE660E diesel engines stand out as stalwarts in industrial applications, owing to their superior performance, longevity, and cost-effectiveness. While previous research has explored diverse combustion strategies for diesel engines, many necessitate intricate modifications to injection systems.

## 2 Materials and methods

Numerical simulations and engine design were performed using AVL-FIRE software. Various process factors, including fuel injection quantity and timing, environmental conditions, and fuel characteristics, were adjusted in response to changes in engine loads and speeds ranging from idle to rated speed. This paper examines the effects of fuel quantity and injection timing on output variables such as ignition delay and engine performance parameters such as power, torque, combustion chamber pressure, fuel consumption, and emission indicators.

**Fig. 1:**
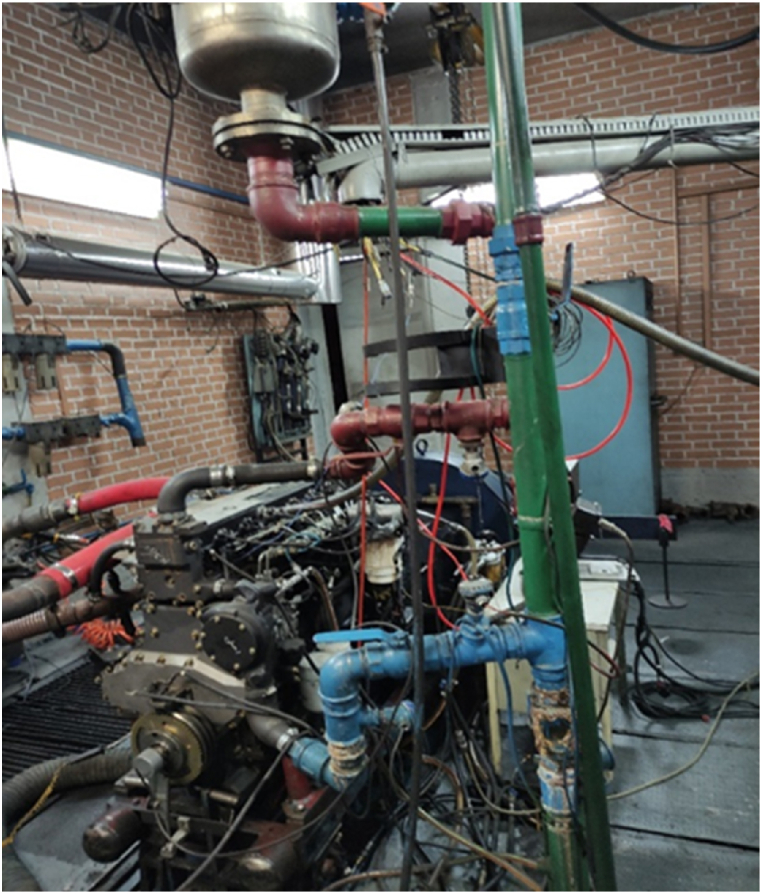
MTE660E engine installed on dynamometer.

The tests related to this study were conducted at the Engineering Research Unit of Motorsazan Company, a subsidiary of Tabriz Tractor Manufacturing Companies. The latest design of diesel engine of a heavy-duty tractors (MTE660E) manufactured by Tabriz Motorsazan Co., was selected as the test engine. The technical specifications and engine installation are shown in Table (1) and Figure (1), respectively.

**Table 1:** Technical specifications of diesel engine MTE660E.

Engine characteristics	Value
Number of cylinders	6
Cylinder bore	100 mm
Stroke	127 mm
Connecting rod length	217 mm
Fuel nozzle pressure	1600 bar
Compression ratio	17.5
Maximum torque	480 N m @ 1600 RPM
Maximum power	89.5 kW @ 2000RPM

### 2.1 Test process

**Fig. 2:**
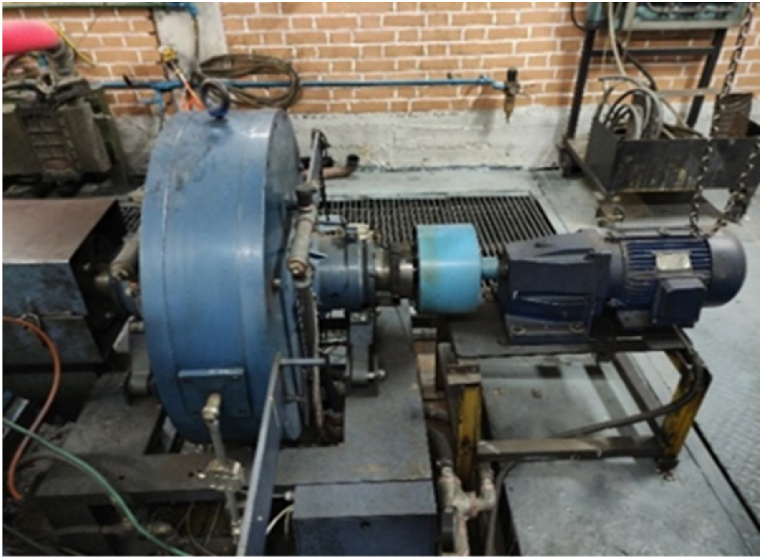
Magnetic dynamometer.

In a controlled test procedure, a magnetic dynamometer was used to control the speed and load of the MTE660E motor. The dynamometer was used to measure and control the braking force and torque of the output shaft. In addition, a 500 kg load cell sensor was used to measure the torque on the reaction shaft. The dynamometer used in the test room at the Motor Research Centre is a 400 kW electromagnetic type ±2N.m accuracy and can maintain a constant engine speed with ±5rpm accuracy (Fig. 2).

**Fig. 3:**
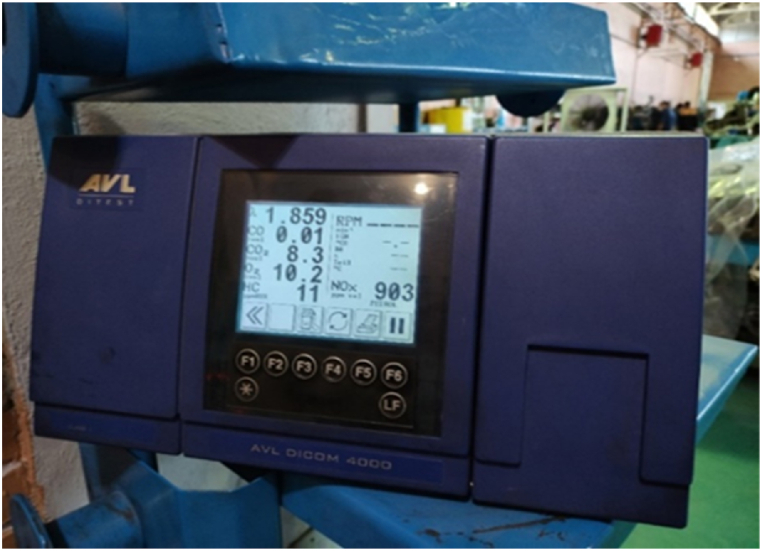
AVL Dicome 4000 exhaust gas analysis device.

In this study, an AVL Indi module 621 piezoelectric pressure sensor was used to determine in-chamber pressure. In addition, Sensors were positioned at certain places in engine to measure water, oil and gas temperatures and the data was recorded and stored by a central computer to manage the engine performance. A mass-based fuel measurement procedure was used to measure fuel consumption that calculated the fuel consumption rate in grams per time step. The fuel tank was placed on a precise digital scale with an accuracy of 0.1 g to record the fuel mass at regular intervals. A hot-wire flow meter was used to control fuel injection systems of engine. During the engine performance test, the emission quantity was measured with an ±5ppm accuracy maximum via an AVL DiCom 4000 Emission Analyzer (Fig. 3) capable of measuring the carbon monoxide (CO), nitrogen oxides (NOx) and unburnt hydrocarbons (UHC) based upon the European emission standards. In addition, smoke opacity monitoring was provided using an AVL Smoke Meter (AVL 415S). The various sensors were used to gauge the CO2, O2, fuel spray angle, oil temperature and all data were sent to the base computer via PLC port. The Dynamometer was applied to perform load and speed tests of The MTE660E diesel engine placed in the engine testing room. Before starting the engine performance test, the engine was first run for approximately half an hour at dynamometer full load and fully warmed up to obtain a stable engine operation. The tested engine injector was equipped with both needle lift sensor and injection timing sensors measuring precision injection timing and exact fuel quantity.

**Fig. 4:**
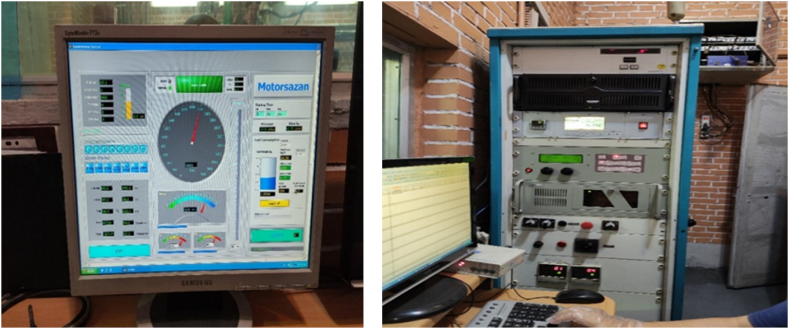
Data recorded by monitor from the measurement sensors.

**Fig. 5:**
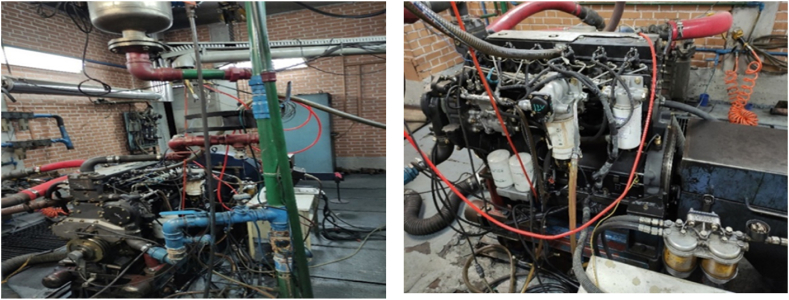
The test room.

A constant braking force is applied to control vehicle speed (Three various engine speeds) at rated motor speed and measure the engine performance and emission parameters. All processes was repeated triple to minimize measurement error. Fig. 4, Fig. 5 show the data recording monitor and the engine testing room, respectively.

#### 2.1.1 Uncertainty analysis

A series of tests were conducted to evaluate the variation of ignition timing at an engine speed of 2000 rpm with an ignition timing (advance) of 32 crank angle before TDC and at full load. Due to differences in heating values and oxygen content among the fuels tested, comparisons were made at the same engine brake mean effective pressure (load) rather than at the air/fuel ratio. The tests also considered the accuracy of the measurements, as shown in the table below, which shows the accuracy of the measurements and the uncertainty of the calculated results. Each test measured volumetric fuel consumption, exhaust smokiness, and regulated exhaust emissions such as nitrogen oxides (NOx), carbon monoxide (CO), and total unburned hydrocarbons (HC). Specific fuel consumption were calculated from the measurements using sample density and net calorific value. Table 2 provides details of the accuracy of the measurements and the uncertainty of the calculated results for various parameters.

**Table 2:** Accuracy of the measurements and the uncertainty of the calculated results.

Measurement	Accuracy
HC	±2 ppm
Time	±0.5 %
Speed	±5 rpm
Soot density	±1 mg/m3
NOx	±5 ppm
CO	±3 ppm
Torque	±0.2 N m
Fuel volumetric rate	±1 %
Power	±1 %
Specific fuel consumption	±1.5 %

### 2.2 Numerical simulation

In this paper, Numerical simulation of diesel fuel combustion was performed in AVL FIRE software to model laminar flame propagation, Kinetics of pollutants formation and flow gradients in the combustion chamber.

### 2.3 Simulation procedure

**Fig. 6:**
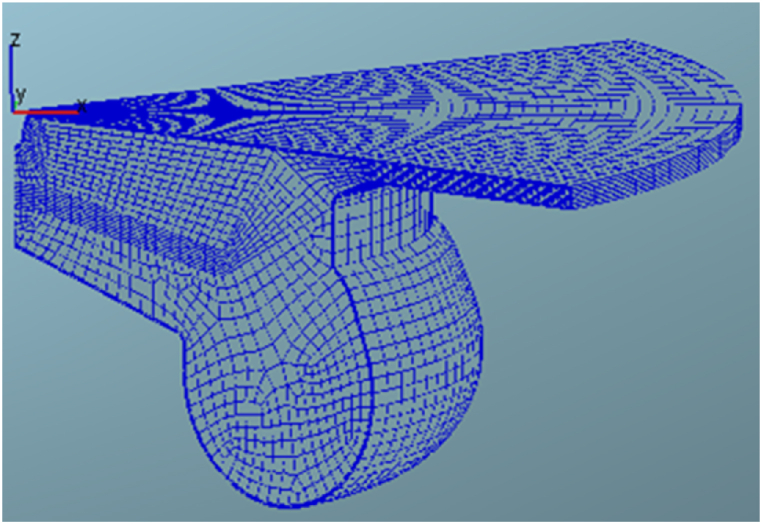
A three-dimensional network of the combustion chamber of an internal combustion engine at top dead center (TDC).

The AVL Fire and solving the mass and energy conservation equation discretized on the basis of a finite volume method was employed for 3D CFD modelling in the combustion chamber, while the computational mesh used for the engine simulations. A SIMPLE algorithm is then generalize to establish a general relationships among fuel pressure, flow, and density in diesel engine. For, it should be created an initial model. The spatial simulation initial conditions from 3-D model of the engine's combustion chamber was carried out to obtain in-cylinder flow simulation, combustion process and operating modes of internal condition of a test engine geometry. Since this research is focused on the MTE660E diesel engines having symmetrical piston cups, a cross section (90-degree) of the cylinder around the vertical axis was considered. The next step is to create a two-dimensional mesh design of the combustion chamber where rotates around vertical axis of the cylinder to model the fully 3D meshes of the entire geometry of combustion chamber (as shown in Fig. 6). The dynamic mesh motion was then achieved for two compression and expansion phases which take place in a closed work loop between the IVC (inlet valve closing) timing to the EVO (exhaust valve opening) timing.

**Fig. 7:**
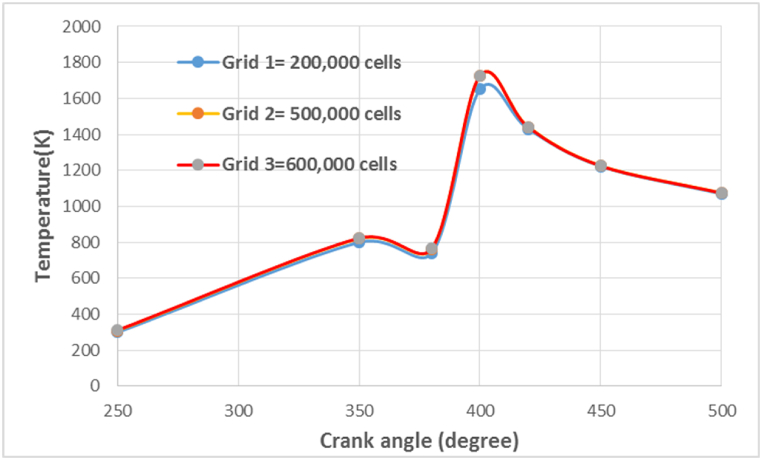
Grid independence for in-cylinder temperature versus crank angle.

When designing a computational model, it is important to ensure that the results obtained are independent of the number of cells used in the computation. This is called grid independence (see Fig. 7). Grid independence is achieved by changing the number and size of the cells and observing the effect on the calculated results. The goal is to find an appropriate number of cells that provides an accurate solution while minimizing computational cost. As shown in the figure, the difference in temperature changes between grid 1 and 2 and 3 is about 9 %. However, the difference between grid 2 and 3 is less than 1 %, indicating that the selected cell size (about 500,000) is appropriate for this study. Balancing computational cost and accuracy is critical in CFD problems, and grid independence analysis is an essential step in ensuring the accuracy of the results obtained.

### 2.4 Initial and boundary conditions

At the end of the intake stage (IVC), the calculated data of combustion chamber volume, air temperature, air pressure, air flow and fuel compositions and quantity were imported into the statistical software. The input variables shown in Table 3 were considered as the numerical simulation initial conditions for a diesel engine. Given the assumed initial state based on estimated parameters, the next step is to use them for subsequent analyses as the combustion phasing shifts from one stage to another in the duty cycle. The simulation initial conditions are presented in Table 3.

**Table 3:** The simulation initial conditions.

Initial condition	value
Start of combustion crank angle degree (intake valve closing crank angle)	149.9°CA
End of combustion crank angle degree (exhaust valve closing crank-angles)	129.5°CA
Initial air pressure value	1.8 Bar
Initial air temperature value	299K
Initial fuel temperature value	290K

For simulation the fluid flow during work, in-cylinder turbulence intensity can be regarded as equivalent to 1.1 of engine speeds. The diesel engine was run at various operating speed of 750 rpm, 1600 rpm, and 2200 rpm. In the present study, the common neat diesel fuel of heavy-duty engines provided by Tabriz oil refinery (Iran) utilized as a common fuel in diesel engines. Moreover, the boundary conditions for the engine CFD 3D model is presented in three different sets to be compared with a real flow situation in actual engine cases. For modelling the engine boundaries based on real flow situation, 3-D model was developed for a combustor condition, while keeping the boundary conditions constant for different operating points. In particular, simulation boundary conditions can be determined as follow.

1. Simulations were performed assuming zero boundary velocity components inside and at the edge of the boundary layer, where the air temperature was held constant at 525 K.
2. It was known that the head boundary involved constant mass flow-inlet to wall. The second boundary set, modeled by a linear-like condition, basically consists of assuming a constant temperature of 450 K with velocity components of zero along the wall.
3. The other set was applied in the piston with boundary condition motion under predefined initial conditions of a constant temperature of 550 K.

### 2.5 Combustion equations

In terms of CFD modeling, the attention will be focused exclusively on closed cycle conservation flows for turbulent and compressible combustion flows to solve governing equations, i.e. the Mass, momentum and energy conservation equations, as shown in Equations (1), (2), (3), respectively [35].

(1) $$\frac{\partial \rho }{\partial t}=-\frac{\partial }{\partial x_{j}}\left(\rho U_{i}\right)$$

(2) $$\begin{array}{l} \rho \frac{DU_{i}}{Dt}=\rho \frac{\partial U_{i}}{\partial t}+\rho U_{i}\frac{\partial U_{i}}{\partial x_{j}}=\rho g_{i}+\frac{\partial \sigma_{ij}}{\partial x_{j}}=\rho g_{i}-\frac{\partial P}{\partial x_{j}}+\\ \frac{\partial }{\partial x_{j}}\left[\mu \left[\frac{\partial U_{i}}{\partial x_{j}}+\frac{\partial U_{j}}{\partial x_{i}}-\frac{2}{3}\frac{\partial U_{k}}{\partial x_{k}}\partial_{ij}\right]\right] \end{array}$$

(3) $$\begin{array}{l} \rho \frac{DH}{Dt}=\rho \left[\frac{\partial H}{\partial t}+U_{j}\frac{\partial H}{\partial x_{j}}\right]=\rho q_{j}+\frac{\partial P}{\partial t}+\\ \frac{\partial }{\partial x_{i}}\left(\tau_{ij}U_{j}\right)\frac{\partial }{\partial x_{j}}\left(\lambda \frac{\partial T}{\partial x_{j}}\right) \end{array}$$

Where, ρ ($kg/m^{3}$) is density, U (m/s) the fluid velocity, g (g) the gravitational acceleration, σ the stress tensor, P(pa) the fluid pressure, μ (pa.s) the fluid viscosity, δ the unit tensor, H (kJ/kg) the fluid static enthalpy, λ heat transfer coefficient, and τ is the fluid shear stress.

### 2.6 Combustion model

In this research, the combustion model was based on the 3-zone extended coherent flame model (ECFM-3Z), investigated in a CFD software AVL FIRE. In addition, the ECFM-3Z (3-Zones Extended Coherent Flame Model) is a combustion model that used to cover all combustion modes (turbulent mixing, auto ignition, propagation flame and diffusion flame). After auto-ignition, the mixing of (cold) fuel and hot oxidizer occurs in the auto-ignition premixed zone, followed by a turbulent diffusion flame instantaneously formed on the borderline of the high temperature region burning the fuel left by this rich trailing flame.

### 2.7 Analysis of exhaust emissions

#### 2.7.1 Mechanism for producing NO

In fuel poor and relatively rich flames, the extended Zeldovich mechanism is responsible to predicts NO generation from atmospheric nitrogen in combustion systems [36]:

(4) $$\begin{array}{l} O+N_{2}↔NO+N\\ N+O_{2}↔NO+O\\ N+OH↔NO+H \end{array}$$

As is seen from (5), (6), the relationships in (61) determines that the NO and N concentration changes for this case is:

(5) $$\begin{array}{l} \frac{d\left[NO\right]}{dt}=k_{1}^{+}\left[O\right]\left[N_{2}\right]+k_{2}^{+}\left[N\right]\left[O_{2}\right]+k_{3}^{+}\left[N\right]\left[OH\right]-\\ k_{1}^{-}\left[NO\right]\left[N\right]-k_{2}^{-}\left[NO\right]\left[O\right]-k_{3}^{-}\left[NO\right]\left[H\right] \end{array}$$

(6) $$\begin{array}{l} \frac{d\left[N\right]}{dt}=k_{1}^{+}\left[O\right]\left[N_{2}\right]-k_{2}^{+}\left[N\right]\left[O_{2}\right]-k_{3}^{+}\left[N\right]\left[OH\right]-\\ k_{1}^{-}\left[NO\right]\left[N\right]+k_{2}^{-}\left[NO\right]\left[O\right]+k_{3}^{-}\left[NO\right]\left[H\right] \end{array}$$

Using these, as the thermal NO is assumed to be zero, and combining of two above equations, equation (7) can be obtain as:

(7) $$\frac{d\left[NO\right]}{dt}=2(k_{1}^{+}\left[O\right]\left[N_{2}\right]-k_{1}^{-}\left[NO\right]\left[N\right]$$

Thus, the equilibrium condition for the above equations can be written in the following form:

(8) $$\begin{array}{l} R_{1}=k_{1}^{+}{\left[O\right]}_{e}{\left[N_{2}\right]}_{e}=k_{1}^{-}{\left[N\right]}_{e}{\left[NO\right]}_{e}\\ R_{1}=k_{2}^{+}{\left[N\right]}_{e}{\left[O_{2}\right]}_{e}=k_{2}^{-}{\left[NO\right]}_{e}{\left[O\right]}_{e}\\ R_{3}=k_{3}^{+}{\left[N\right]}_{e}{\left[OH\right]}_{e}=k_{3}^{-}{\left[NO\right]}_{e}{\left[H\right]}_{e} \end{array}$$

The mechanism can be written for each of the main reactions:

(9) $$\beta =\frac{\left[NO\right]}{{\left[NO\right]}_{e}}\Rightarrow \left[NO\right]=\beta {\left[NO\right]}_{e}$$

Considering the equilibrium concentrations of O, OH, N2 and O2 and the kinetic concentrations of N and NO, the equations can be written as:

(10) $$\begin{array}{l} \frac{d\left[N\right]}{dt}=0.0\Rightarrow \left[N\right]=\\ \frac{k_{1}^{+}{\left[O\right]}_{e}{\left[N_{2}\right]}_{e}+k_{2}^{-}{\left[NO\right]}_{e}{\left[O\right]}_{e}+k_{3}^{-}{\left[NO\right]}_{e}{\left[H\right]}_{e}}{k_{2}^{+}{\left[O_{2}\right]}_{e}+k_{3}^{+}{\left[OH\right]}_{e}+k_{1}^{-}{\left[NO\right]}_{e}} \end{array}$$

(11) $$\begin{array}{l} \frac{d\left[NO\right]}{dt}]2\left\{R_{1}-\beta R_{1}\frac{R_{1}+\beta \left(R_{2}+R_{3}\right)}{\beta R_{1}+R_{2}+R_{3}}\right\}=\\ 2R_{1}\left[\frac{1-\beta^{2}}{1+\frac{\beta R_{1}}{R_{2}+R_{3}}}\right] \end{array}$$

The emission of NO can be calculated using the equilibrium concentrations of combustion products, temperature, pressure, and fuel equivalence ratio for each region.

### 2.8 Mechanism for soot formation

The mechanism of producing soot is based on a system of different equations solved to calculate the soot number density defined by means of nucleation and coagulation in an elementary volume [37]. In addition, the soot mass fraction ($S_{\varphi s}$) source term is described by using the following semi-empirical equation includes nucleation, surface growth and oxidation terms (12).

(12) $$S_{\varphi s}=S_{n}+S_{g}+S_{O_{2}}$$

The mass fraction ($\varphi_{s}$) of soot outlined above is obtained by solving differential equation that also gives rise to a source term in the ($S_{\varphi s}$) equation. The first reaction step ($S_{n}$) responsible for soot particle nucleation can be written schematically as (13):

(13) $$S_{n}=C_{n}\;\mathrm{EXP}\left\{\frac{-{\left(f-f_{n}\right)}^{2}}{\sigma_{n}^{2}}\right\}$$

The particle coagulation (S_g_) is assumed to be soot surface growth on the surface of the particles:

(14) $$S_{g}=A.F\left(f,\varphi_{s}\right).P^{0.5}\;\mathrm{EXP}\left(-\frac{E_{a}}{RT}\right)$$

Where, the $E_{a}$ is activation energy, the R (J/mol.K) universal Gas Constant, the P (bars) pressure, the T temperature (K), and the ($F\left(f,\varphi_{s}\right)$) is the function of effective viscosity of soot surface growth.

Soot nucleation involves oxidation including surface reactions of soot with acetylene, hydroxyl radical and molecular oxygen, the chemical coalescence of PAH on the particle surface, coagulation features the collision and union of particles (soot nucleation) are also soot surface growth. Thus this step is in the present study written as:

(15) $$S_{O_{2}}=-F\left(\varphi_{s},P_{O_{2}},T\right)$$

Where $P_{O_{2}}$ is the partial pressure of oxygen.

### 2.9 Mechanism for producing CO

The producing CO mechanisms, semi-empirical Heywood model (1988) are the principal chemical reactions which generate pollutant carbonic oxide, in combustion systems. The first step is chemical attachment of oxygen to the surface (absorption), followed by desorption of the oxygen with the attached fuel component from the surface as a product. (16):

(16) $$\begin{array}{l} CO+OH↔CO_{2}+H\\ 2CO_{2}+O↔CO+O_{2} \end{array}$$

It is assumed that reactions (16) are reversible, in which case forward rates of both reactions is nearly balanced by the rate of its reverse at typical flame front temperatures. Therefore the concentration of CO can potentially be in equilibrium, as discussed in Ref. [38]. Oxidation of soot particles is considered a two-stage process. It has been assumed that CO is oxidized to form CO2 exclusively at typical flame temperatures. Therefore, the first term of equation is an absorption process of oxygen by the surface, followed by desorption of the oxygen with the attached fuel component from the surface, In this case, the rate of CO producing is proportional to:

(17) $$\begin{array}{l} \frac{d\left[CO\right]}{dt}=K_{1}^{-}{\left[CO_{2}\right]}_{e}{\left[H\right]}_{e}+K_{2}^{+}{\left[CO_{2}\right]}_{e}{\left[O\right]}_{e}-\\ K_{1}^{+}{\left[CO\right]}_{e}{\left[OH\right]}_{e}-K_{2}^{-}{\left[CO\right]}_{e}{\left[O_{2}\right]}_{e} \end{array}$$

Using the following relations:

(18) $$\begin{array}{l} R_{1}=K_{1}^{+}{\left[CO\right]}_{e}{\left[OH\right]}_{e}=K_{1}^{-}{\left[CO_{2}\right]}_{e}{\left[H\right]}_{e}\\ R_{2}=K_{2}^{+}{\left[CO_{2}\right]}_{e}{\left[O\right]}_{e}=K_{2}^{-}{\left[CO\right]}_{e}{\left[O_{2}\right]}_{e}\\ C=R_{1}+R_{2}\\ D=\frac{R_{1}+R_{2}}{{\left[CO\right]}_{e}} \end{array}$$

Under these assumptions, the CO emissivity coefficient are calculated at initial temperatures of 2800 K and constant pressures ranging from 15 to 40 atm. accordingly, the CO emissivity coefficient is computed as:

(19) $$\begin{array}{l} \frac{d\left[CO\right]}{dt}=R_{1}+R_{2}-\left[CO\right]\frac{R_{1}+R_{2}}{{\left[CO\right]}_{e}}\\ \frac{d\left[CO\right]}{dt}=C-D\left[CO\right] \end{array}$$

## 3 Results and discussion

In this research, a numerical investigation on the engine performance, in-cylinder combustion, and emissions was performed in a MTE660E heavy duty diesel or compression ignition (CI) engine used in the ITM1500 heavy-duty tractor manufactured by Tabriz Motorsazan Co. a 3D CFD simulations were performed by AVL Fire software and CFD results were compared with the experimental results of the MTE660E engine. The engine performance, heat release rate, ignition delay, mechanism of soot, CO and NOx emission producing were investigated numerically. According to the obtained results, it can be understood from both experimental and CFD studies that could give significant improvements in overall the parameters of engine performance and emissions with a good balance between them.

### 3.1 Model validation

**Fig. 8:**
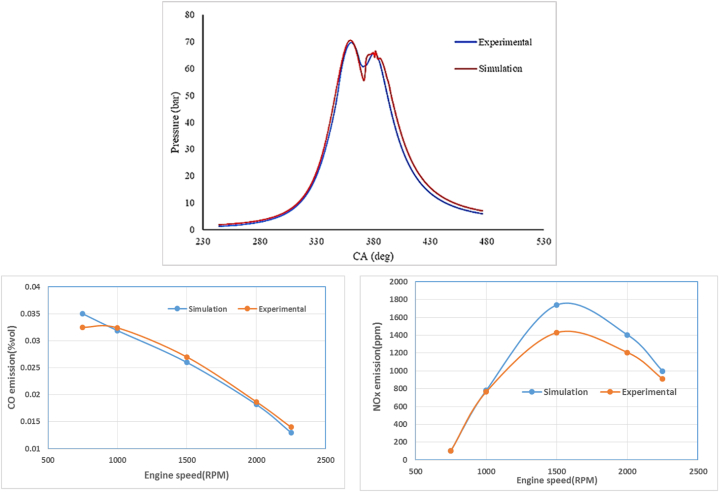
Validation of simulation results with in-cylinder pressure, CO and NOx emission.

In this research, simulation results were compared and validated with the experimentally validated predictions of models. As shown in Fig. 8, Models validation was acceptable on 1/4 cylinder compression ignition engine at an operating speeds of 1600 RPM with increases of air/fuel ratio of 1.75 and the in-cylinder pressure could be predicted with accuracy of about 94.4 %. The CFD validation is besides numerical analysis of the proposed model to predict against the performance and emissions data results obtained from an experiment at 21 CA after top dead center in compression ignition (CI) engine.

Fig. 8 also shows a comparison between the results obtained from simulations and experimental data for the emissions of CO and NOx as a function of engine speed. There is a slight difference between the two sets of results, which indicates that the accuracy of the presented model is quite high. Therefore, we conclude that the model is suitable and effective for predicting the behavior of the engine and its emissions under different operating conditions.

### 3.2 Functional specifications

#### 3.2.1 Braking torque (BE)

**Fig. 9:**
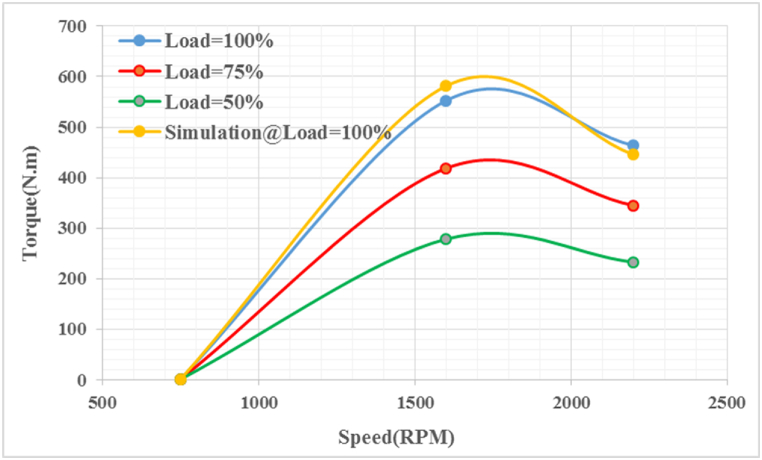
Braking torque at different speeds and loads.

Fig. 9 shows the torque variations at different loads. The results show that at each load level, the engine brake torque increases with increasing engine speed up to 1600 rpm before decreasing. These simulation results can be compared with actual diesel combustion test data to validate the model and emphasize the significant impact of brake torque on engine performance. As a result, the proposed models predict brake torque with a high degree of accuracy at 94.53 %. This conclusion is supported by other researchers [39].

#### 3.2.2 Specific fuel consumption (SFC)

**Fig. 10:**
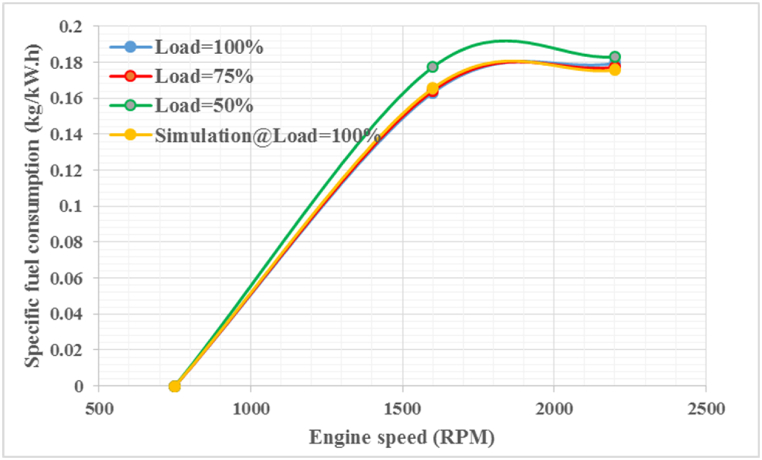
Specific fuel consumption at different speeds and loads.

Fig. 10 shows the specific fuel consumption as a function of engine speed for partial loads (50 % and 70 %), full load engine operation, and simulation load. The results show that at each engine load level, the specific fuel consumption increases as the engine speed decreases. Specifically, the highest SFC value is observed at 2200 rpm and 50 % load, while the lowest SFC value occurs at 1750 rpm and 100 % load. To ensure the validation and reliability of the simulation model, statistical comparisons were made between experimental and simulated engine fuel consumption results. The results show that the AVL model accurately predicts the SFC, achieving an efficiency of 98 % with an error of less than 1.83 %. Similar results were observed for other fuels and operating conditions [39].

#### 3.2.3 Exhaust gas temperature (EGT)

**Fig. 11:**
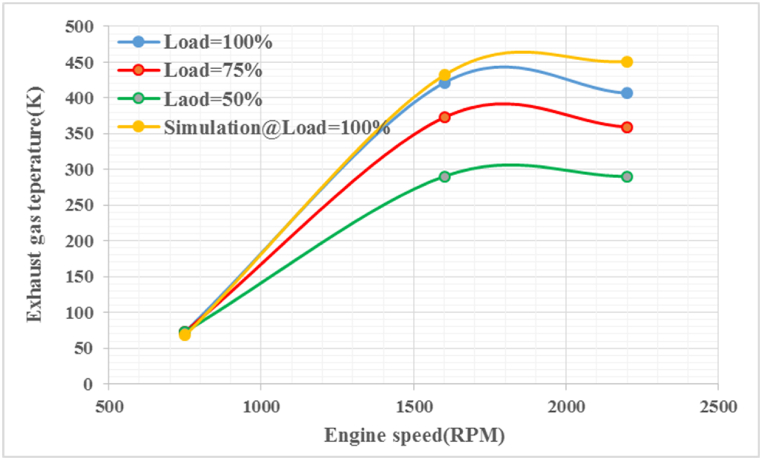
The temperature of exhaust gases at different speeds and loads.

Fig. 11 illustrates the variations in exhaust gas temperature with respect to engine speed for load variables of 50 %, 100 %, and full load (100 %), in comparison with the simulated load. As evidenced by the graph, there is a clear correlation between engine speed and EGT. The highest EGT value is observed at an engine speed of 1600 rpm under 100 % load, while the lowest SFC is observed at 750 rpm and 50 % load. The AVL model's prediction for engine EGT was validated, demonstrating good agreement between predicted and experimental values (with errors consistently below 6.66 %). This indicates that the AVL model effectively captures the relationship between independent variables and dependent variables, including exhaust gas temperature. It is noteworthy that ignition delay and combustion characteristics in compression ignition engines play a crucial role in reducing exhaust gas temperature [40].

#### 3.2.4 CO emission

**Fig. 12:**
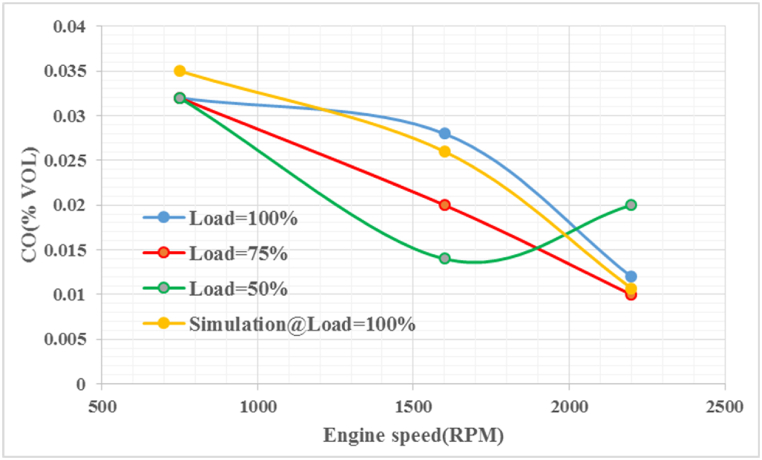
CO emission at different speeds and loads.

Looking at Fig. 12, it can be seen that the calculated results for CO emissions for both the 75 % and 100 % load models, except for the 50 % load, match the slope of the corresponding 100 % simulation load curve as engine speed increases. The data indicate that CO emissions decrease as engine speed increases for 50 % and 100 % loads. The relationship between engine speed and CO emissions in the enhanced combustion processes is generally consistent that higher engine speeds result in lower CO emissions due to the increased effectiveness of the oxygen content of the fuel in reducing CO at higher speeds. Validation of the models showed acceptable results, with CO emissions predicted with a lower error rate of approximately 9.47 %. It is important to note that CO emissions decrease with total oxygen concentration, which can be affected by combustion timing and other factors.

#### 3.2.5 HC and NOx emissions

**Fig. 13:**
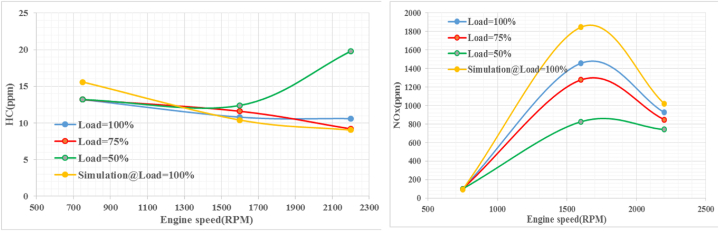
HC and NOx emissions at different speeds and loads.

Fig. 13 shows that the emission of hydrocarbon pollutants begins to decrease as the engine speed increases at all load levels except 50 %. The peak HC emission occurs at 19.5 mm, where the load is at its lowest value of 50 % at an engine speed of 2200. The phenomenon of higher engine speeds resulting in lower HC emissions for a given load can be attributed to improved combustion efficiency at higher engine speeds. Unburnt hydrocarbons typically result from incomplete combustion, uneven fuel distribution, and suboptimal ignition system conditions [41]. The p-values obtained, which are greater than 5 %, indicate that there are no significant differences between the simulated and experimental values. Therefore, the results generated by AVL Fire can be considered reliable. It is known that small hydrocarbon diffusion typically leads to nitrous oxide emissions. In Fig. 13, NOx emissions are plotted against engine speed at different engine loads (25 %, 75 %, and 100 %) compared to the simulated load. The graph shows an increase in NOx emissions as the engine speed increases, followed by a decrease. Notably, the simulated load shows the highest growth rate and maximum NOx emission. The peak NOx emission is observed at 1600 rpm under full load. The lowest NOx levels are recorded at 50 % load and 2200 rpm. Nitrogen oxides (NOx) are formed in the combustion chamber of diesel engines due to several factors, including high temperatures, oxygenated environments, and combustion duration.

The fuel-air mixture in the combustion chamber of a diesel engine is compressed and heated to high temperatures, which allows the nitrogen and oxygen molecules in the fuel to react with each other to form NOx. The higher the temperature, the greater the amount of NOx produced. The presence of oxygen in the combustion chamber of a diesel engine promotes the formation of NOx. The more oxygen present, the more NOx is produced. The longer the combustion time, the more time the nitrogen and oxygen molecules have to react, resulting in more NOx production.

To minimize NOx production in a diesel engine, several methods have been implemented, including increasing the amount of exhaust gas recirculation, using a Selective Catalytic Reduction (SCR) system, and modifying the combustion chamber geometry.

Notably, lower NOx emissions are observed when the engine is operated at higher speeds at lower loads due to the presence of more unreacted oxygen and the increase in both volumetric efficiency and flow velocity resulting in less residence time available for NOx formation. Therefore, NOx emissions decrease with engine speed for a given load. This occurs due to decrease in residence time of high temperature burning gas in the combustion chamber resulting in lower NOx emissions. Other Similar studies have been done to investigate NOx emission with increasing engine speed on internal combustion engines and lower NOx was observed when the engine is operated at higher engine speed [42]. According to all the validation results, it was observed signiﬁcant difference between the actual and the predicted values (P value < 0.05). It can be stated that the AVL model has a good ability to predict the pollutant emissions of the engine in terms of independent variables. So that the results are useable and can be predicted.

#### 3.2.6 Soot emission (SE)

**Fig. 14:**
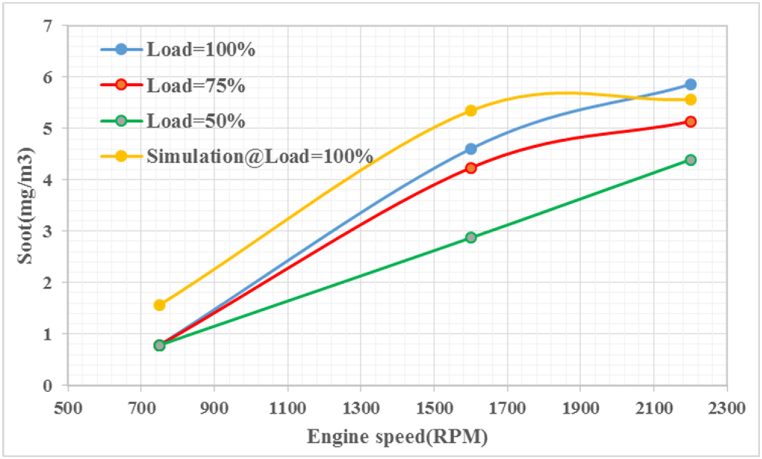
Soot emission at different speeds and loads.

Researches have implemented various strategies to reduce NOx such as catalyst and EGR [43,44], but most of these scenarios lead to higher soot, Therefore soot analysis along with NOx at different engine speeds are needed. Studies show that distribution of soot particle size depends on engine load [45]. Fig. 14 shows that the soot emissions at three engine loads (50, 75, and 100 % (full load)), as well as the simulation load, increased as the engine speed increased. The results showed the highest soot emissions at 100 % load and 2200 rpm, and the lowest soot emissions at 50 % load and 750 rpm. The diesel engine at high speed appears to have slightly more soot formation, especially at higher engine loads, due to the shorter mixing time compared to low speed [46]. This is mainly due to the challenge in the air/fuel mixing process, resulting in a higher amount of unreacted oxygen and excess air concentration in the exhaust gas during cold start, which causes the increase in SE with engine speed. The model validation showed the satisfactory results against the available experimental data for soot emission perdition with a low error of 5 %.

### 3.3 Optimizing the diesel engine performance parameters using CFD methods and a regression model

#### 3.3.1 Peak chamber temperature (PCT)

**Fig. 15:**
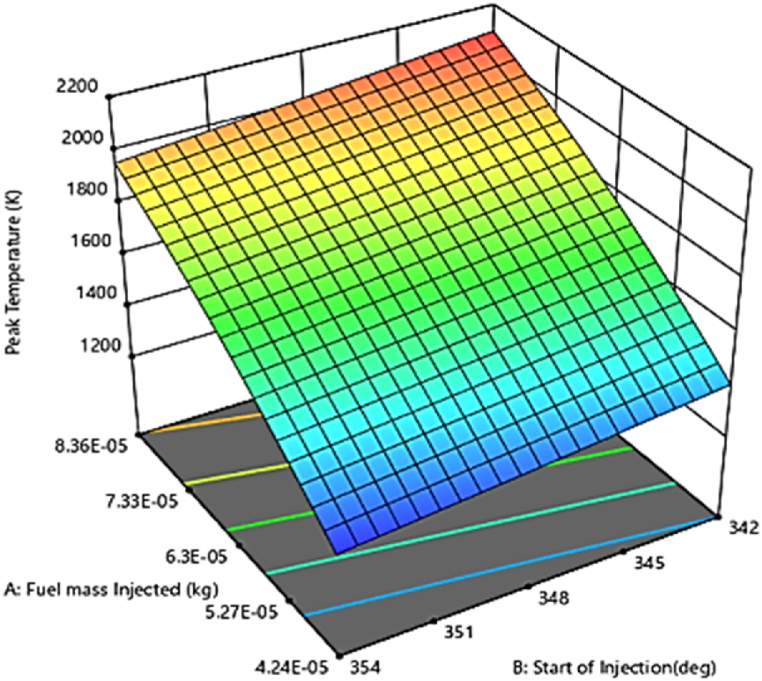
Effect of injecting time and fuel injection quantity on peak chamber temperature.

**Fig. 16:**
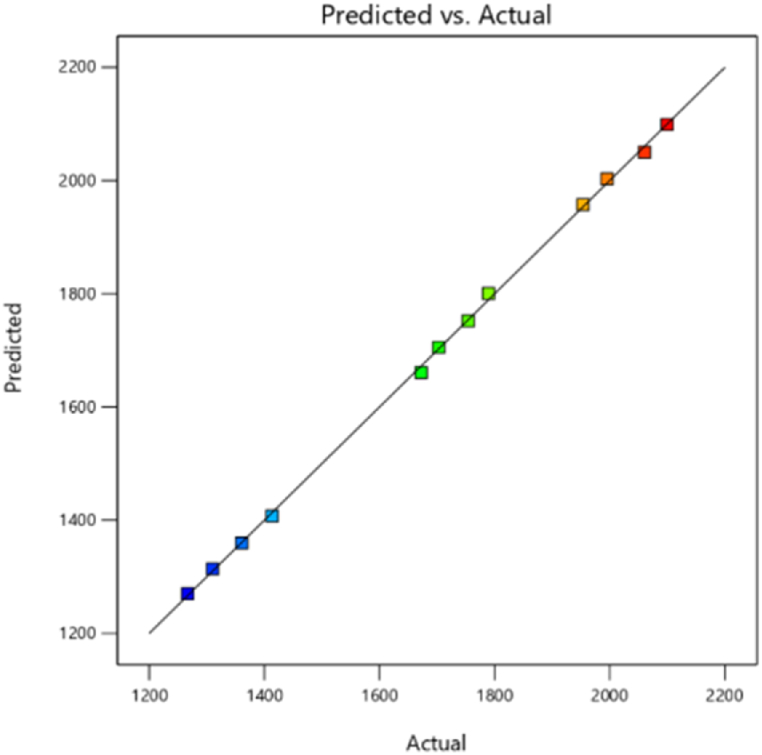
Prediction model of the peak chamber temperature.

A numerical simulation, including complex three-dimensional (3D) computational fluid dynamics (CFD), is developed to model the combustion chamber condition and optimize the performance parameters of a heavy-duty diesel engine using design expert software. As the validation showed, the prediction results of the AVL model can be reliable, through which the 3D response surface plots for dependent variables with respect to independent variables were presented. Fig. 5 shows the peak chamber temperature as a function of fuel injection quantity and injection time. As can be seen in Fig. 14, the highest PCT is obtained from a crank angle degree of 342°CA with an injection quantity of $8.36\times 10^{-5}$ kg. Combustion chamber temperature is affected by many factors, including gas temperatures inside the combustion chamber, compression ratio, exhaust gas temperature, intake port temperature, injection timing, and vaporization and combustion characteristics. The plot in Fig. 15 shows that the peak chamber temperature decreases with fuel injection delay, but increases with increasing fuel quantity. This could be attributed to reduce the ignition delay resulted in achieving a homogeneous mixture and increase the combustion pressure and temperature under stoichiometric conditions [47]. Other Similar studies have been conducted to optimize the peak chamber temperature [48]. The regression model in Fig. 16 shows a comparison between the predicted and actual relevant output of peak chamber temperature. According to all the results, the model was found to have a high prediction accuracy, resulting in an improvement in the prediction capability for the peak chamber temperature with a greater R^2^ of 90 % and less than 5 % error.

#### 3.3.2 Brake mean effective pressure (BMEP)

**Fig. 17:**
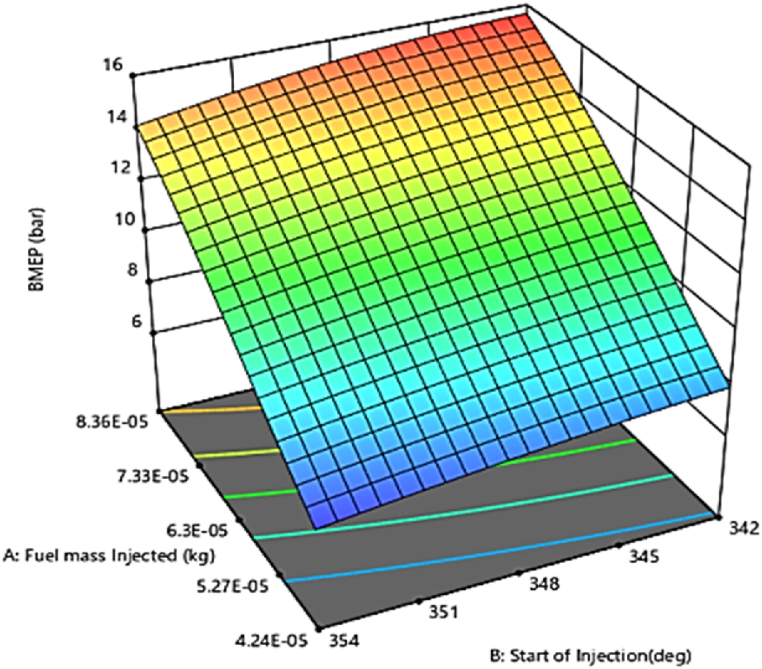
Effect of injecting time and fuel injection quantity on brake mean effective pressure.

**Fig. 18:**
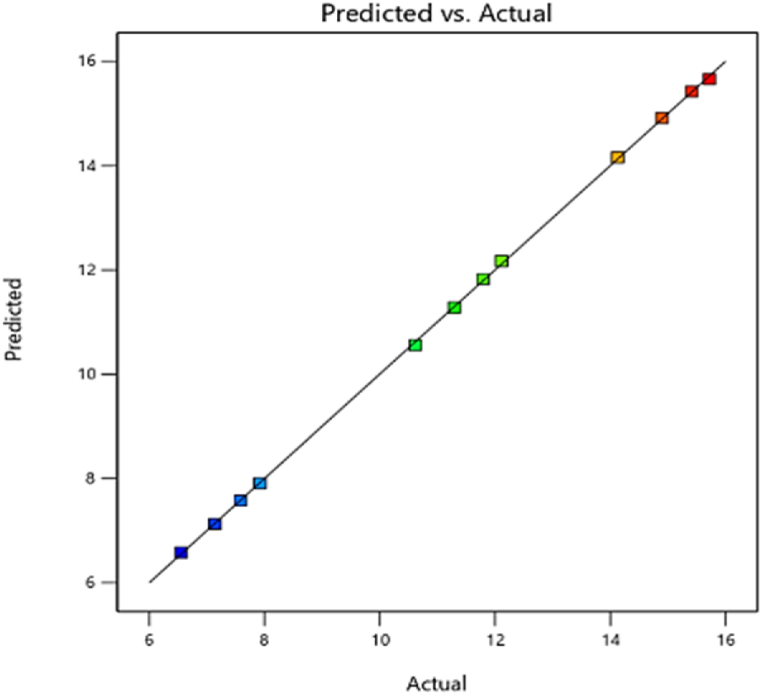
Prediction model of brake mean effective pressure.

In addition to peak chamber temperature, brake mean effective pressure was an important parameter in the diesel engine performance study. The investigated diesel engine operating conditions covered certain variations of the brake mean effective pressure that were related to the brake power measurement. This means that brake mean effective pressure is essential for predicting brake performance [49]. The peaks of the brake performance are based on the highest value of the brake mean effective pressure (BMEP) at the given engine speed. Fig. 17 represents the interaction effects of fuel injection quantity and injecting time on brake mean effective pressure variabe in the 3D surface plots using RSM. The optimum (maximum) value of brake mean effective pressure was obtained with an injection timing of 342 °CA (crank angle degree) and a fuel quantity of $8.36\times 10^{-5}$ kg. Fig. 18 shows the regression model for predicting the average effective pressure of the diesel engine, which is very accurate. This model is based on the optimization results and effectively predicts the average effective pressure based on various input variables. The comparison between the predicted and actual CFD results in Fig. 17 shows that the model has a high ability to predict the performance parameters of the engine in terms of brake mean effective pressure variables with an acceptable accuracy (R^2^> 90 %).

#### 3.3.3 Engine torque

**Fig. 19:**
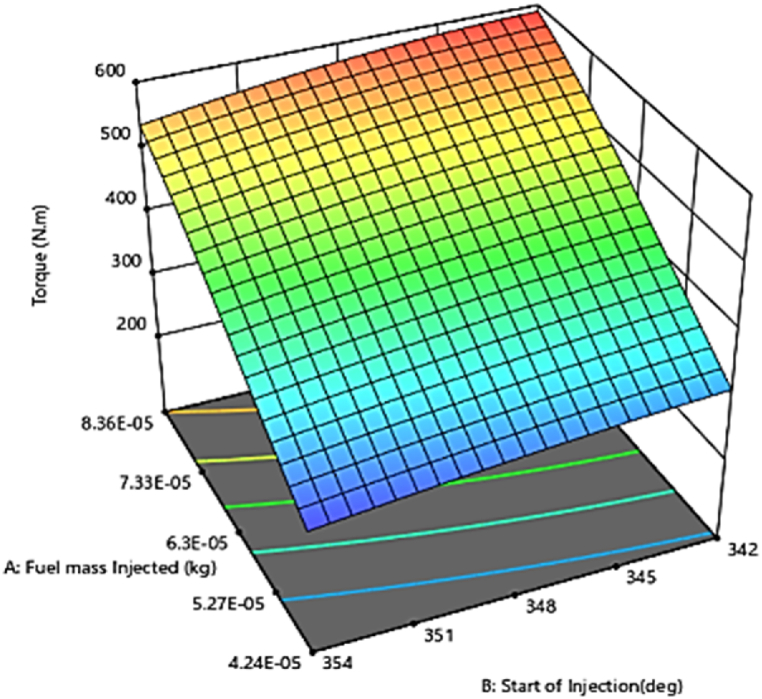
Effect of two parameters, injecting time and fuel injection quantity, on torque.

**Fig. 20:**
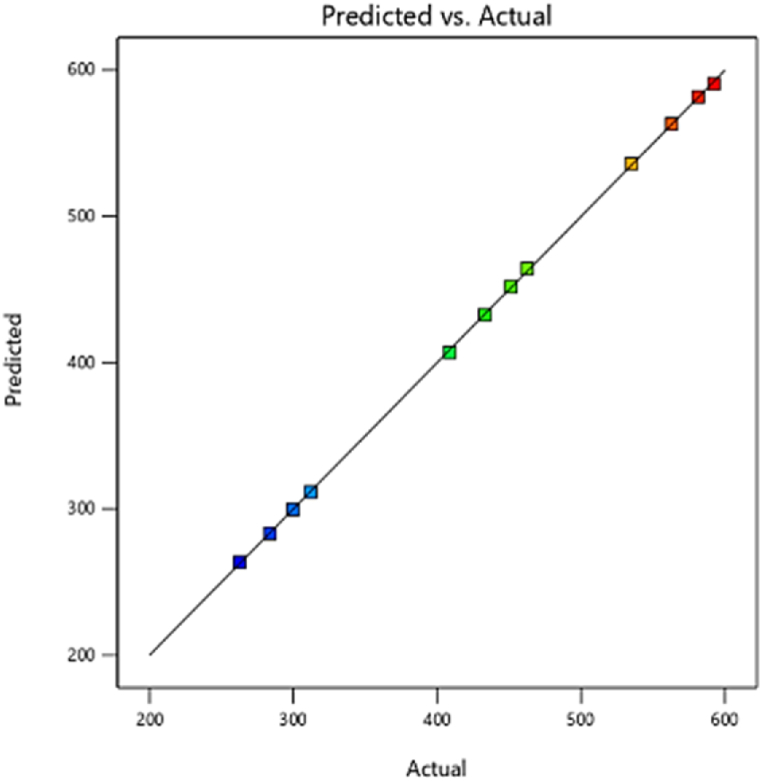
Prediction of torque performance parameter.

Fig. 19 shows the combined effect of injection timing (crank angle) and fuel injection quantity on the torque variables in three-dimensional response surface plots. As can be seen from the plot, increasing the fuel injection quantity and the early injection time results in an increase in torque. Fuel injection with the correct delay (with respect to the crank angle) at the exact preset time in the engine cycle results in an acceptable fuel-air ratio followed by complete combustion, increased power and torque, higher thermal efficiency, and smooth running of the engine [50]. The prediction results related to the regression model development of the diesel engine torque showed that the accuracy of prediction and generalization ability of the AVL model is acceptable (R^2^> 90 %) (Fig. 20). Researchers have reported similar results regarding the of fuel injection timing on the combustion performance and emissions of a compression ignition engine. The results showed that advancing the injection timing increased the ignition delay and played a significant role to optimize the engine performance. This research confirms that maximum torque and pressure was achieved at an optimum injection timing of 342°b TDC [51].

#### 3.3.4 Engine thermal efficiency (FTE)

**Fig. 21:**
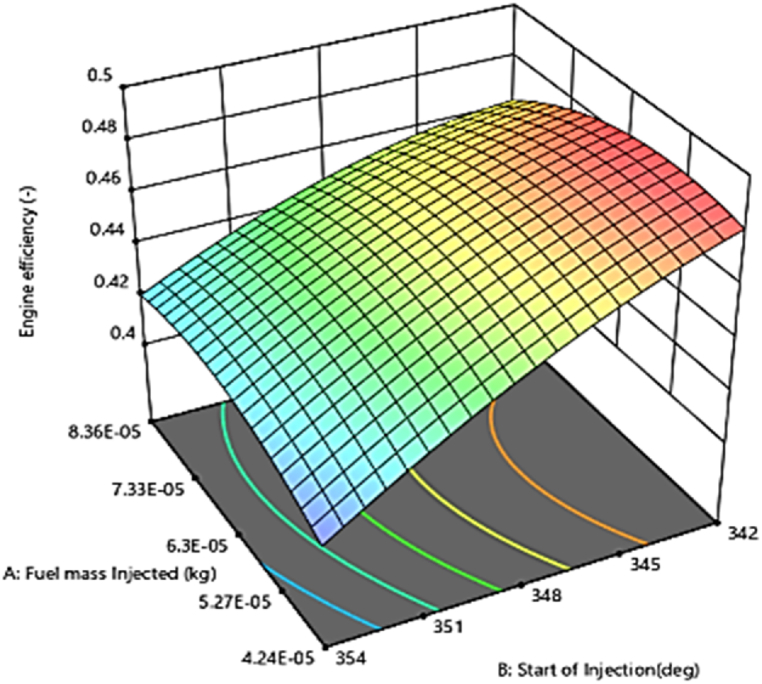
Effect of injecting time and fuel injection quantity on engine efficiency.

**Fig. 22:**
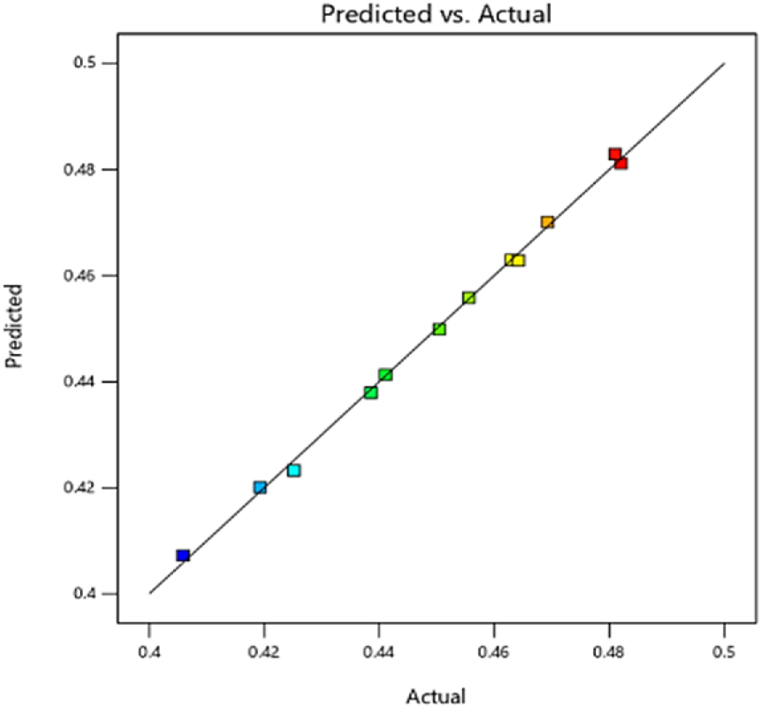
Prediction of engine efficiency Functional parameter.

A 3D plot of engine thermal efficiency versus fuel injection quantity and injection times using response surface methodology is shown in Fig. 21. It is known that the longer ignition delay causes incomplete combustion, resulting in low thermal efficiency [52]. The changes in FTE, always trend upward with injecting time, although the highest amounts of thermal efficiency were obtained at an injection time of 345° CD and with an injection quantity of $6.3\times 10^{-5}$ kg. This combination of injection parameters is recommended based on the results of this research. The regression model developed for corresponding engine thermal efficiency is presented in Fig. 22, which are in good agreement between the actual and predicted values (R^2^> 90 %).

#### 3.3.5 Emission of NOx

**Fig. 23:**
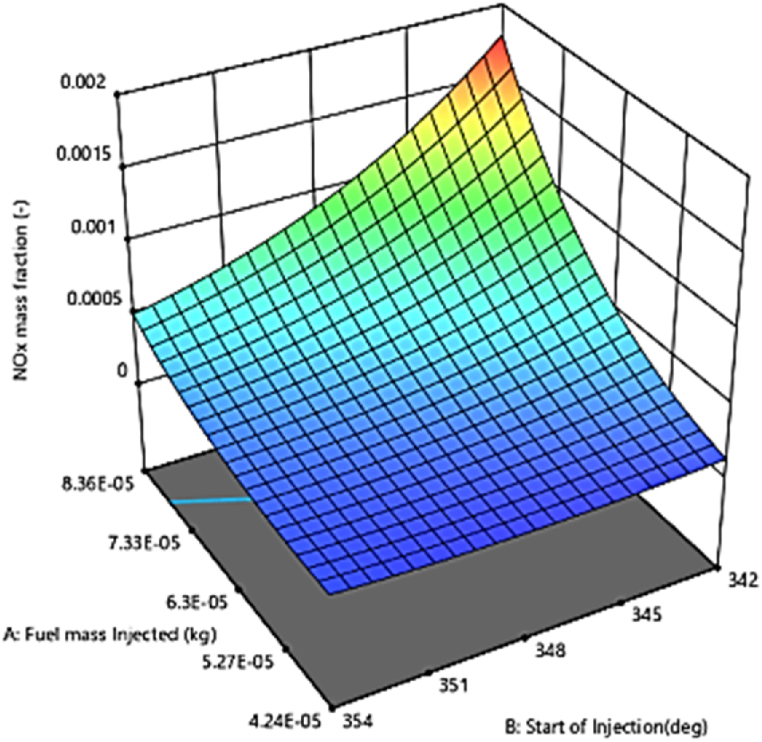
Effect of the two parameters injection time and injection fuel quantity on NOx emission.

**Fig. 24:**
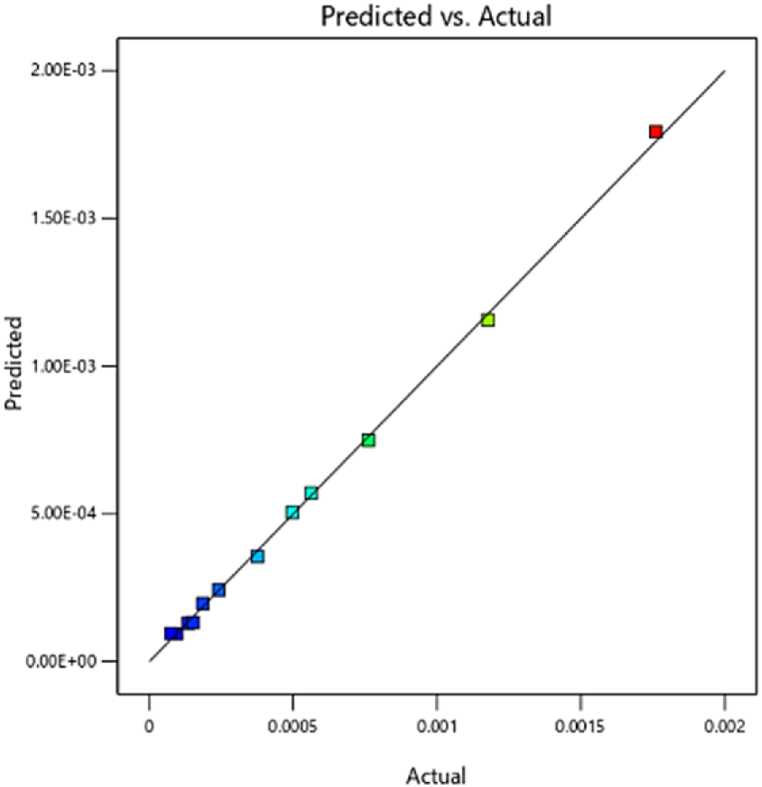
Prediction of the NOx mass fraction.

Fig. 23 shows the combined effect of injection timing and fuel injection quantity on NOx emission in three-dimensional response surface plots. The research result showed that for a given fuel injection quantity, ignition delay at an injection time of 354 DC can provide much lower in-cylinder pressure and temperature, resulting in reduced NOx emission compared to the ignition time of 342 DC. This could be attributed to the higher ignition delay resulting in over-injection of fuel into the combustion chamber prior to the self-ignition of the fuel particles. In conclusion, the high level of produced fuel/air ratio causes the excessive exhaust smoke and NOx emission [53]. The in-cylinder temperature and pressure drop by delaying the start of injection timing (changed from 342° to 354° b TDC) can reduce the oxidation of soot and improve the engine performance. Fig. 24 shows a regression model for comparing predicted and measured NOx emissions based on response surface methodology, which provides evidence of the reliability of the measurements. As can be seen in the model, good predictive ability can be observed to maximize thermal efficiency at lower NOx levels by optimizing the independent variables (error <5 %). Similar results have been reported for injection time effects on NOx emission. The results of this research are consistent with previous studies, including that of Agarwal et al. [34], who investigated the effect of fuel injection time and pressure on combustion, greenhouse gas emissions, and performance characteristics of a diesel engine. The results indicated that increasing fuel injection time reduced CO2 and HC emissions, but increased NOx emissions [54,55].

## 4 Conclusions

In this paper, a 3D CFD simulation model based on AVL FIRE software was used to estimate the peak chamber temperature, brake mean effective pressure, engine torque, engine thermal efficiency, and emissions at various injection times with different fuel injection quantities. The research results showed that.

1 The recommended injection time of 342 °C and the specific fuel quantity lead to a better combustion efficiency, resulting in high engine performance and low emissions.
2 The specific fuel consumption was highest at 2200 rpm with 50 % load, while the lowest specific fuel consumption was observed at 1750 rpm with 100 % load.
3 Maximum NOx emissions were observed at full load.
4 Optimization of injection timing and fuel injection quantity can effectively enhance the performance of the MTE660E engine, improving combustion efficiency and power, and reducing emissions without requiring expensive overhaul.
5. The peak chamber temperature decreases with fuel injection delay, but increases with increasing fuel quantity. The optimum (maximum) value of brake mean effective pressure was obtained with an injection timing of 342 °CA and a fuel quantity of $8.36\times 10^{-5}$ kg.
6. Increasing the fuel quantity and the early injection time results in an increase in torque. The optimum (maximum) value of brake mean effective pressure was obtained with an injection timing of 342 °CA and a fuel quantity of $6.3\times 10^{-5}$ kg.
7. The AVL model was validated against experimental data and, the error between the measured and calculated value of combustion characteristic and emissions was acceptable (R^2^> 90 %), Error <5.6 %).

The AVL model accurately predicts engine brake torque, specific fuel consumption, and exhaust gas temperature with high accuracy and low error margins, effectively capturing the relationship between engine speed, load, and performance metrics. These findings support the model's use in optimizing engine performance. In order to achieve the emission goals without compromising performance further researches need to be implemented. Therefore, open cycle diesel simulation to study the impact of intake and exhaust valve timing on the performance, along with energy and exergy analysis combined with the findings from this study which investigated the optimal timing and injected fuel mass on the efficiency and emissions, can lead to higher efficiency and lower emissions of diesel engines.

## Additional information

No additional information is available for this paper.

## Data availability statement

Data will be made available on request.

## CRediT authorship contribution statement

**Zuhair Aldarwish:** Writing – review & editing, Writing – original draft, Visualization, Validation, Software, Resources, Methodology, Investigation, Funding acquisition, Formal analysis, Data curation, Conceptualization. **Mohammad Hossein Aghkhani:** Writing – review & editing, Writing – original draft, Supervision, Project administration. **Hassan Sadrnia:** Writing – review & editing, Writing – original draft, Supervision, Project administration, Methodology. **Javad Zareei:** Writing – review & editing, Writing – original draft, Supervision, Project administration.

## Declaration of competing interest

The authors declare that they have no known competing financial interests or personal relationships that could have appeared to influence the work reported in this paper.
